# Neurodevelopment in patients with repaired tetralogy of Fallot

**DOI:** 10.3389/fped.2024.1137131

**Published:** 2024-04-26

**Authors:** Laura Mercer-Rosa, Emmanuelle Favilla

**Affiliations:** Division of Cardiology, Department of Pediatrics, University of Pennsylvania Perelman School of Medicine, Children’s Hospital of Philadelphia, Philadelphia, PA, United States

**Keywords:** tetralogy of Fallot, neurodevelopment, outcome, child, congenital heart defects

## Abstract

Neurodevelopmental sequelae are prevalent and debilitating for patients with congenital heart defects. Patients born with tetralogy of Fallot (TOF) are susceptible for abnormal neurodevelopment as they have several risk factors surrounding the perinatal and perioperative period. Some risk factors have been well described in other forms of congenital heart defects, including transposition of the great arteries and single ventricle heart disease, but they have been less studied in the growing population of survivors of TOF surgery, particularly in infancy and childhood. Adolescents with TOF, even without a genetic syndrome, exhibit neuro-cognitive deficits in executive function, visual-spatial skills, memory, attention, academic achievement, social cognition, and problem-solving, to mention a few. They also have greater prevalence of anxiety disorder, disruptive behavior and attention-deficit hyperactivity disorder. These deficits impact their academic performance, social adjustment, and quality of life, thus resulting in significant stress for patients and their families. Further, they can impact their social adjustment, employment and career development as an adult. Infants and younger children can also have significant deficits in gross and fine motor skills, cognitive deficits and abnormal receptive language. Many of the risk factors associated with abnormal neurodevelopment in these patients are not readily modifiable. Therefore, patients should be referred for evaluation and early intervention to help maximize their neurodevelopment and improve overall outcomes. More study is needed to identify potentially modifiable risk factors and/or mediators of neurodevelopment, such as environmental and socio-economic factors.

## Introduction

Patients born with tetralogy of Fallot (TOF) are at increased risk for abnormal neurodevelopment (1–3). Some of the risk factors have been well described in other forms of congenital heart defects such as in d-transposition of the great arteries and single ventricle heart disease (3–12). Known patient-related risk factors include those surrounding the pre-natal, neonatal and perioperative period. Here we provide a brief overview of the multiple stages in a patient's exposure to the abnormal physiology from TOF.

## Pre-natally

In fetuses with congenital heart defects, the the brain can have delayed maturation and and is at risk of injury during development secondary to altered oxygen delivery and blood flow (4, 13–16). Studies have demonstrated impaired brain growth and diminished cerebral tissue oxygenation in fetuses with TOF, despite the fact that the combined cardiac output is preserved in fetuses with TOF, indicating that obstruction of the right ventricular outflow tract in TOF can also result in deleterious re-distribution of the fetal circulation (17, 18). Moreover, the presence of a genetic syndrome, present in up to one fifth of patients born with TOF, poses additional risk for abnormal neurodevelopment (discussed into further detail below) (1, 19–25).

## In the neonatal period

Factors such as prematurity, low birth weight and exposure to cyanosis can affect neurodevelopment (26, 27). *Surgical* risk factors include exposure to cardiopulmonary bypass, deep hypothermic circulatory arrest (less commonly used in TOF surgical repair), temperature regulation and cerebral perfusion during bypass (28). The preferred approach to the cyanotic neonate with TOF remains a subject of debate (29–32). When performed in the neonatal period, complete surgical repair for TOF resolves cyanosis, but exposes the patient to the aforementioned risks associated with surgical repair (33). Greater and longer exposure to anesthetic agents in the neonatal period is associated with peri-operative brain injury and lower cognitive scores in children tested at 12 months of age (34). Conversely, a staged-approach including a palliative procedure in the neonatal period prolongs cyanosis and carries its own risks. Overall, either approach is acceptable for standard risk cyanotic neonates (32).

Post-operatively, most patients with TOF recover from surgery and are discharged within about a week after repair, however there is a subset that experiences a complicated post-operative course with arrhythmias, low cardiac output, seizures, prolonged mechanical respiratory support and hospitalization, all of which can pose additional risk for abnormal neurodevelopment (35–38). To our knowledge, the impact of post-operative restrictive physiology of the right ventricle in the post-operative period on future neurodevelopment has not been investigated. However, restrictive physiology is a marker for worse post-operative clinical course after TOF repair, which in turn is associated with worse neurovelopment, as shown by our group and others (39–41). Other post-operative factors can influence brain development, many of which remain understudied, including social and environmental factors, and associated cardiac and non-cardiacco-morbidities (41). In summary, risk factors for abnormal neurodevelopment in patients with TOF exist and are multifactorial. While many are not readily modifiable, their recognition and appropriate patient referral for testing and early-intervention are paramount.

## Neurodevelopment in infancy and early childhood

Neurodevelopment has been investigated in children and adolescents with TOF. In particular, the group led by Prof. Hövels-Gürich in Germany studied neurodevelopment in children with TOF compared to those with ventricular septal defects and to healthy children (42–45). Their goal was to compare neurodevelopment and exercise capacity in children born with a cyanotic defect to those with a heart failure-related defect [ventricular septal defects (VSD)]. They assessed several domains of neurodevelopment, including language and gross motor skills, intelligence (intelligence quotient), and academic performance. Exercise capacity was evaluated using questionnaires (45). The TOF group had greater motor function deficits as compared to those with VSDs, and these deficits were correlated with other outcome measures including neurologic deficits, lower IQ and impaired language skills. In fact, they subsequently fared worse on oral and speech motor control functions as well as on oral and speech apraxia tests (42). Notably, peri-operative risk factors were not associated with neurodevelopment at this age, including pre-operative severity of cyanosis in the TOF group, which is generally regarded as a risk factor. Conversely, in a subsequent analysis these investigators identified significant deficits in various domains of attention which were more pronounced in patients with TOF as compared to those with VSDs and healthy controls. They postulate that pre-operative hypoxemia is deleterious to the oxygen-sensitive areas in the brain responsible for attention control, including the prefrontal cortex and striate body (43). Interestingly, this group of TOF children had comparable behavioral and quality of life measurements to those with VSDs and normal children (44).

In 2007, a group from Belgium led by Marijke Miatton investigated the intellectual, neuropsychological and behavioral functioning in children at around age 8 years. They were compared to children with acyanotic heart disease and to normal controls. While children with TOF tested similarly to those with acyanotic heart disease, they showed worse intellectual abilities and a less favorable neuropsychological profile as compared to healthy children without heart defects. Specifically, they had lower IQ, worse language and sensorineural function. To exemplify, children with TOF had greater difficulty to quickly respond to verbal commands of increased difficulty and also had lower motor speed. It is possible that these deficts (which can be subtle) explain why children with TOF perform worse academically, as suggested by their parents' questionnaires (46).

We had the opportunity to conduct a single-center retrospective cohort study of infants and children with repaired TOF that underwent clinically based neurodevelopmental assessment at the Children's Hospital of Philadelphia Cardiac Kids Developmental Follow-up Program (CKDP). We included patients that underwent TOF repair between 0 and 12 months of age and that were participants in a prospective cohort study (47). Patients were evaluated between 0 and 4 years of age at the CKDP. Those with genetic syndromes were included, and data analysis was adjusted for presence of a genetic syndrome. First, we described the neurodevelopmental status in this group and identified socioeconomic, patient, and medical management factors associated with neurodevelopment and with referral for early intervention therapy. Next, we conducted sub-analyses to test the association between screening tests administered in infancy (Bayley Infant Neurodevelopmental Screener [BINS] and the Peabody Developmental Motor Scale (PDMS) with the Bayley Scales of Infant and Toddler Development, Third Edition (Bayley-III), which is a more definitive test of neurodevelopment performed after one year of age (48, 49). We also investigated socioeconomic factors using the patient's census block group as a proxy for the neighborhood based on the geocoded home address at the time of the first CKDP evaluation and calculated a social disorganization index in block group level (41). We considered an array of possible risk factors as exposures, including pre-operative (birth history, cardiac anatomy, pre-operative use of prostaglandin, palliative procedures prior to TOF repair), operative, and post-operative [cardiopulmonary bypass (CPB) duration, number of CPB runs, lowest pH and temperature during CPB, post-operative complications, and length of hospital stay] factors. Genetic syndromes were categorized as 22q11.2 deletion syndrome, trisomy 21 and other syndromes. We used longitudinal data analysis methods to test the association between each exposure and the neurodevelopment test as the main outcome, adjusting for the presence of genetic syndrome.

We found significant gross motor deficits in almost half of the patients and deficits in receptive language, cognitive, and fine motor skills, which were seen in one-third of patients. We also found that the median composite scores on the PDMS and Bayley-III tests were lower than the normative population's expected scores and particularly lower in patients with trisomy 21 and 22q11.2 deletion syndrome.

We reported that several neighborhood-level factors related to poverty were associated with greater odds of abnormal neurodevelopment, most notably on the early screening BINS evaluation. Complex cardiac anatomy (presence of aortopulmonary collaterals and TOF with atrioventricular canal), longer duration of cardiopulmonary bypass, and greater number of cardiac and non-cardiac post-operative complications were associated with worse neurodevelopment outcome across the various tests administered. Interestingly, duration of hospitalization after TOF repair was not associated with neurodevelopment outcomes in this group.

When examining factors associated with referral to early intervention, we showed that TOF repair in the neonatal period, history of prostaglandin utilization prior to TOF repair, and use of deep hypothermic circulatory arrest during TOF repair were significant, indicating practitioner's awareness of potential risk factors for abnormal neurodevelopment. In parallel, patient's discharge home after birth (to be readmitted later for TOF repair) was associated with lower odds of referral.

In summary, we identified significant deficits in children with repaired TOF which were detected in infancy and showed that an abnormal early screening test was associated with lower scores after 1 year of age. In fact, the early BINS when used in babies with other morbidities, such as prematurity predicts neurodevelopmental deficits later in childhood (22, 23). Further, abnormal results in the PDMS in neonates and infants might suggest risk of future fine and gross motor deficits, as suggested by our sub-analysis. We also bring to light associations between socioeconomic factors with neurodevelopment in childhood, indicating that the social environment needs to be taken into account when risk-stratifying these children.

Our findings emphasize the importance of neurodevelopment testing in patients with TOF starting in infancy, similarly to what has become a standard of care for patients with transposition of the great arteries and hypoplastic left heart syndrome (5, 7, 21).

We have recently conducted a study in a small group of children with repaired TOF tested at age 5–6 years (before entering grade school). They underwent testing with the Wechsler Preschool and Primary Scale of Intelligence Fourth Edition (WPPSI-IV), the Bracken School Readiness Assessment Third Edition (BSRA-3), and the Beery-Buktenica Developmental Test of Visual Motor Integration Sixth Edition (VMI). Parents completed the Behavioral Rating Inventory of Executive Function Preschool Version (BRIEF-P). Although there were no significant deficits in this small group of 18 children, the overall Z scores were below 0 (and above 0 for the BRIEF-P, where lower scores are better), and were associated with patient morbidiy, including number of specialists seen (unpublished data). These findings reiterate the importance of testing these patients and testing early such that appropriate therapies are put in place.

In summary, neurodevelopmental deficits are prevalent in survivors of TOF repair but could be modified if identified and treated in early childhood so that patients can maximize their neurocognitive potential during adolescence and adulthood. Future avenues for research and quality improvement should include the development of outreach efforts to ensure adequate resource allocation and access to care for those patients found to be at highest risk, based on both initial neurodevelopment screening and socioeconomic factors, starting in early infancy.

## Neurodevelopment in adolescents and young adults with TOF

Neurodevelopment has been understudied in patients with TOF, despite this being a prevalent and growing population of survivors of heart surgery (50). Adolescents with TOF, even without a genetic syndrome, test lower than their peers in several neuro-cognitive domains, including executive function, visualspatial skills, memory, attention, academic achievement and social cognition. Further, adolescents with TOF have significant deficits in problem-solving and visual-spatial memory (51). They also have greater prevalence of anxiety disorder, disruptive behavior and attention-deficit hyperactivity disorder (52). Parents exhibit significant levels of stress when their child has neurobehavioral difficulties, which can negatively impact the child's development into adolescence (53). Therefore, this constellation of neuro-psycho-cognitive morbidities is concerning as they can impact academic performance, social adaptation and quality of life, and lead to challenges in adult life.
